# *Bt* rice in China — focusing the nontarget risk assessment

**DOI:** 10.1111/pbi.12720

**Published:** 2017-04-18

**Authors:** Yunhe Li, Qingling Zhang, Qingsong Liu, Michael Meissle, Yan Yang, Yanan Wang, Hongxia Hua, Xiuping Chen, Yufa Peng, Jörg Romeis

**Affiliations:** ^1^ State Key Laboratory for Plant Diseases and Insect Pests Institute of Plant Protection Chinese Academy of Agricultural Sciences Beijing China; ^2^ College of Plant Science & Technology Huazhong Agricultural University Wuhan China; ^3^ Agroscope Biosafety Research Group Zurich Switzerland

**Keywords:** *Bt* rice, ecosystem services, environmental risk assessment, nontarget effects, surrogate species

## Abstract

*Bt* rice can control yield losses caused by lepidopteran pests but may also harm nontarget species and reduce important ecosystem services. A comprehensive data set on herbivores, natural enemies, and their interactions in Chinese rice fields was compiled. This together with an analysis of the Cry protein content in arthropods collected from *Bt* rice in China indicated which nontarget species are most exposed to the insecticidal protein and should be the focus of regulatory risk assessment.

## Introduction

Rice (*Oryza sativa*) suffers massive yield losses from attacks by a complex of lepidopteran pests (Chen *et al*., 2011). To control these pests, researchers have developed genetically engineered (GE) rice lines that produce insecticidal Cry proteins derived from *Bacillus thuringiensis* (*Bt*) (Cohen *et al*., 2008; Li *et al*., 2016; Liu *et al*., 2016). Before a *Bt* rice line can be cultivated, the risks to the environment must be assessed. This includes the evaluation of potential adverse effects on valued nontarget arthropods (NTAs) and the ecosystem services they provide (Devos *et al*., 2015; Romeis *et al*., 2008). This premarket NTA risk assessment requires information about which species live in the receiving environment and which species are most likely exposed to the Cry proteins (Romeis *et al*., 2013, 2014; Todd *et al*., 2008). We have therefore conducted a comprehensive literature search to identify (i) the taxonomic groups and species of aboveground arthropods present in the rice‐growing regions of Central and Southern China, and (ii) the known food web interactions of those species. Furthermore, we have (iii) collected arthropods in a field experiment with *Bt* rice to assess the level of plant‐produced Cry2A‐protein to which the NTA species are exposed. Based on this information surrogate species for laboratory toxicity studies to support the regulatory risk assessment of *Bt* rice are suggested.

## Results and discussion

### Aboveground rice arthropods

**Figure 1:**
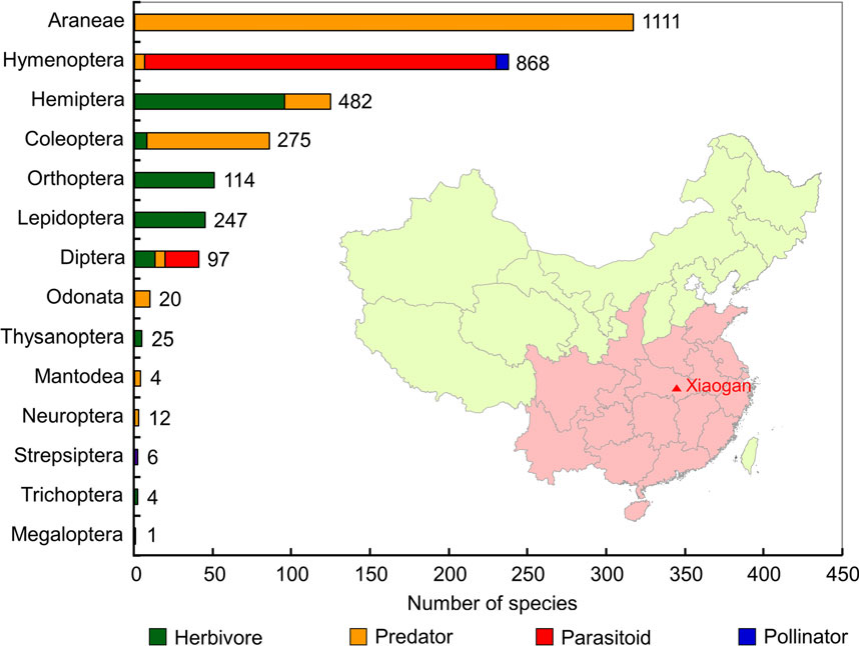
Numbers of arthropod species (sorted by order) recorded in the rice‐planting regions of Central and Southern China (area indicated in red). Total numbers of records available in the published literature are indicated beside the bars. The red triangle represents Xiaogan where arthropods were collected in a Cry2A‐transgenic rice field. Map has been adapted from http://d-maps.com/carte.php?num_car=17501&lang=en.

The literature search identified 201 publications that contained relevant information on aboveground arthropods present in rice fields in Central and Southern China. From those publications, a total of 3266 records were retrieved for 930 arthropod species belonging to 14 taxonomic orders and four functional groups (Figure 1; Table S1). Of the 930 species, 23.7% were represented by herbivores (26.3% of records), 49.0% by predators (45.9% of records), 26.5% by parasitoids (27.5% of records) and 0.9% by pollinators (0.3% of records). The group with most species and records was spiders (Araneae), which represented 34.1% of the species (and 34.0% of the records); all spiders are predators. The second most species‐rich and abundant group was the Hymenoptera (25.6% of species, 26.6% of records), which contains parasitoids, predators and pollinators, followed by the Hemiptera and Coleoptera, which constituted 13.4% and 9.2% of the total number of arthropod species, and 14.8% and 8.4% of records, respectively. The species belonging to those two orders are either herbivores or predators. Orthoptera (5.5% of species) and Lepidoptera (4.8%) are herbivores. Diptera (4.4%) are mainly herbivores, while seven predator and 21 parasitoid species were identified. The remaining seven orders (Odonata, Thysanoptera, Mantodea, Neuroptera, Strepsiptera, Trichoptera, and Megaloptera) contained only 27 species in total, representing 2.9% of the total number of species (Figure 1). In general, the number of species is linked to the number of records of a particular group. Exception is the Lepidoptera that contained the most important rice pests for which a relatively high number of published records is available.

### Rice arthropod food web

**Figure 2:**
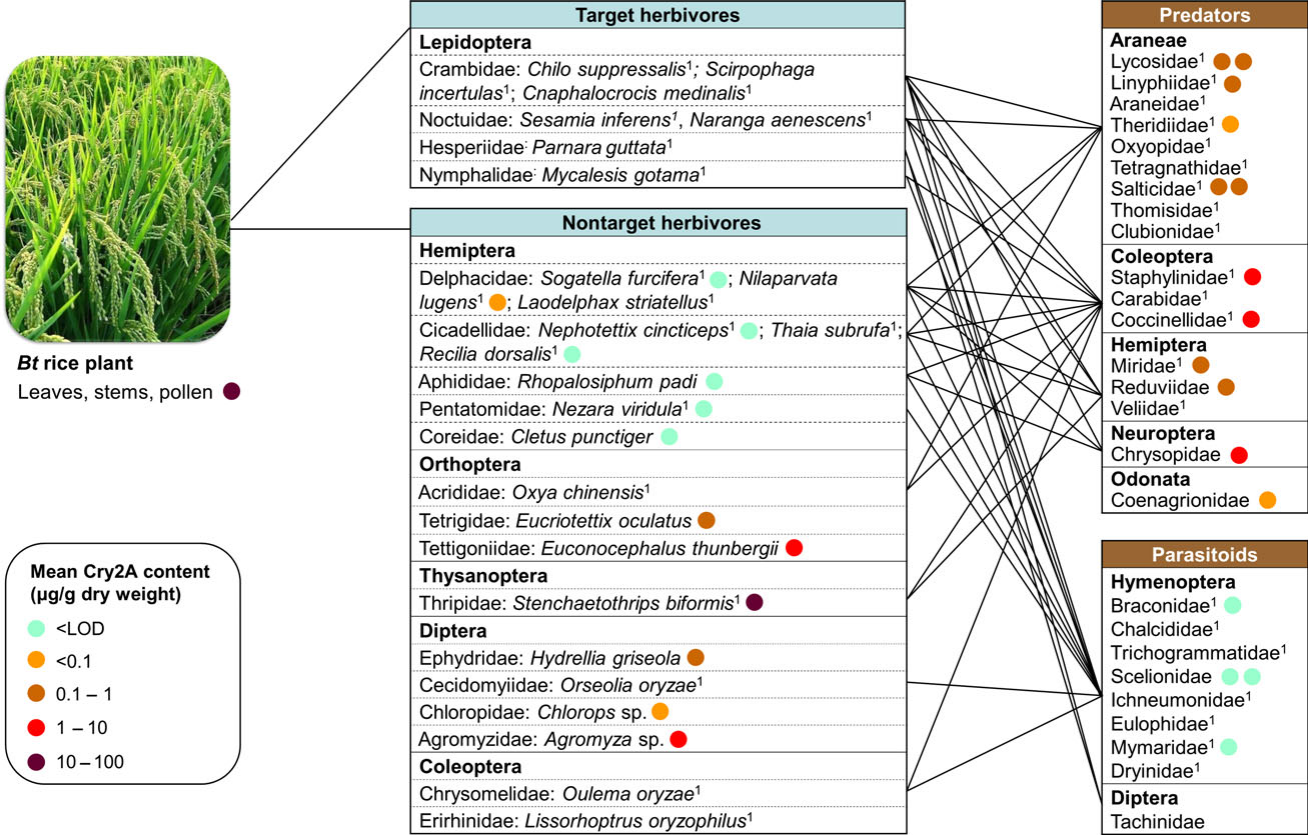
Simplified arthropod food web of rice in Central and Southern China and the content of plant‐derived Cry2A protein in each arthropod species (other than target herbivores). Species are grouped into orders and families. Lines indicate a verified interaction between different families reported in the literature (details are provided in Table S2). For herbivores, only species are listed for which ≥10 records were available (^1^) or that were collected in the field experiment; the latter were analysed by ELISA (coloured circles). For natural enemies, families are listed that contain species with ≥10 records (^1^) or that were represented by species that were collected in the field experiment; the latter were analysed by ELISA (coloured circles). The coloured circles following arthropod species or families indicate the highest Cry2A content measured at any of the sampling dates (one circle represents one species). < LOD = below the limit of detection. Taxa not followed by a coloured circle are commonly reported in the literature but have not been collected in the field experiment.

Published information on the trophic interactions of the most abundant rice arthropods (abundance was roughly estimated based on the number of published records, see Table S1) was used to construct a simplified food web (Figure 2; Table S2). Interactions with natural enemies have been reported for 26 herbivorous species from six orders.

Most reports concern the three most important Lepidoptera pests, that is, *Chilo suppressalis*,* Scirpophaga incertulas*, and *Cnaphalocrocis medinalis* (all Crambidae), which are the targets of *Bt* rice, and the two most important Hemiptera pests, that is, *Sogatella furcifera* and *Nilaparvata lugens* (both Delphacidae).

According to the literature, the major predators of the lepidopteran rice pests belong to ten families of Araneae, and there are other predatory species from the Coleoptera, Hemiptera, and Neuroptera (Figure 2; Table S2). The parasitoids of lepidopteran rice pests are mainly from six families of Hymenoptera with a few species from two families of Diptera (i.e., Tachinidae and Sarcophagidae) (Figure 2; Table S2). In addition to the three major lepidopteran insect pest species, the Lepidoptera *Naranga aenescens* (Noctuidae), *Parnara guttata* (Hesperiidae), *Mycalesis gotama* (Nymphalidae), and *Pseudaletia separata* (Noctuidae) are also recorded as rice pests. Because they do not cause substantial rice losses, however, they are rarely investigated, and little information is available regarding their natural enemies (Table S2).

The natural enemies of the two major hemipteran pests, *S. furcifera* and *N. lugens*, have been extensively studied. Most predator species reported to attack hemipteran herbivores are the same species that attack lepidopteran herbivores (Figure 2; Table S2), because they are mainly generalists. The parasitoids of hemipteran pests belonging to the Delphacidae are mainly from the hymenopteran families Trichogrammatidae, Mymaridae, and Dryinidae (Figure 2). Similarly, hymenopteran parasitoids are known for the plant bug *Nezara viridula* (Pentatomidae) (Table S2). The thrips *Stenchaetothrips biformis* (Thysanoptera: Thripidae) is also a common rice pest in Southern China, and its predators are mainly from the Coleoptera and Hemiptera, while no parasitoid has been recorded (Figure 2; Table S2). Orthopteran species such as *Oxya chinensis* (Acrididae) are also commonly found in rice fields, and their predators mainly include species belonging to the Araneae, Coleoptera, and Mantodea (Table S2). *Oulema oryzae* (Chrysomelidae), an important Coleoptera pest in China, is attacked by coleopteran predators and hymenopteran parasitoids (Figure 2; Table S2). The major natural enemies of dipteran pests are hymenopteran parasitoids (Table S2).

### Exposure of arthropods to Cry2A produced by *Bt* rice

To assess the level at which arthropods are exposed to Cry proteins in *Bt* rice fields, a replicated field experiment was conducted near Xiaogan (Hubei Province, China) in the years 2011 and 2012.

The concentrations of Cry2A detected in rice tissues collected in 2011 and 2012 were similar (Table S3). Rice leaves contained the highest concentrations of Cry2A (from 54 to 115 μg/g DW), followed by rice pollen (from 33 to 46 μg/g DW). The stems contained the lowest concentrations (from 22 to 32 μg/g DW).

Different sampling techniques (including suction sampling, beating sheet and visual searching) were used to collect the 29 most frequently encountered plant‐dwelling arthropod species in the *Bt* and control rice plots during and after anthesis in 2011 and before, during and after anthesis in 2012. The highest measured concentrations of Cry2A in the collected arthropods at any of the sampling dates are indicated in Figure 2.

A total of 13 nontarget herbivores from 11 families belonging to the Hemiptera, Orthoptera, Diptera, and Thysanoptera were collected and analysed (Figure 2; Table S3). In the order Hemiptera adults of *S. furcifera* and nymphs and adults of *N. lugens* contained trace amounts of Cry2A (<0.06 μg/g DW) while the protein was not detected in other species. In contrast, larger amounts of Cry2A (from 0.15 to 50.7 μg/g DW) were detected in all but one sample of the Diptera, Thysanoptera, and Orthoptera. The thrips *S. biformis* contained the highest concentrations of Cry2A of all collected arthropods, which were close to the concentrations in the rice tissues. During anthesis, *S. biformis* contained Cry2A at 51 μg/g DW, which was higher than the concentration in specimens collected before anthesis (35 μg/g DW). Similarly, the protein level in *Agromyza* sp. (Diptera: Agromyzidae) was >2 times higher in samples collected during rice anthesis than before or after anthesis (Table S3). In contrast, the level in *Euconocephalus thunbergii* (Orthoptera: Tettigoniidae) was almost 2.5 times higher in samples collected after anthesis than during anthesis.

Cry2A was estimated in a total of 12 predatory arthropod species belonging to 10 families in five orders (Table S3). Araneae represent the most abundant aboveground predators in the rice ecosystem, and six species from four families were analysed. While spiders collected before or after anthesis generally did not contain measurable amounts of Cry2A, the protein was detected in spiders collected during anthesis at concentrations ranging from 0.02 to 0.57 μg/g DW; the concentrations in spiders were two to three orders of magnitude lower than those in plant tissues (Figure 2; Table S3). All samples of hemipteran species contained Cry2A protein at comparable levels (concentrations ranged from 0.05 to 0.34 μg/g DW). No Cry2A protein was detected in samples of predatory beetles (Coccinellidae and Staphylinidae) collected before rice anthesis. However, the beetles and lacewings (Neuroptera) contained significant amounts during anthesis ranging from 0.20 to 2.60 μg/g DW. These concentrations were one to two orders of magnitude lower than those in plant tissues (Figure 2; Table S3). Adults of four species of parasitoids were collected from three families of Hymenoptera, but Cry2A levels were below the limit of detection (LOD) for all of them (Figure 2; Table S3).

Overall our data show a reduction in Cry protein concentrations from lower to higher trophic levels. This is in accordance with field studies from other *Bt*‐transgenic crops producing different Cry proteins, including maize (Cry1Ab: Harwood *et al*., 2005; Obrist *et al*., 2006; Cry3Bb1: Meissle and Romeis, 2009), cotton (Cry1Ac: Torres *et al*., 2006) and soybean (Cry1Ac: Yu *et al*., 2014).

### Implications for nontarget risk assessment

Risk assessments of *Bt* rice should focus on taxonomic and functional groups that are both common and highly exposed to the produced Cry proteins (Romeis *et al*., 2013). These groups include the common predators in the Araneae, Coleoptera, Hemiptera, and Neuroptera, all of which contained significant amounts of Cry protein in our field experiment. Predators might be exposed to the Cry protein when they consume prey but also when they consume rice pollen or plant sap as a supplemental food source. Elevated Cry2A concentrations during anthesis indicate that several predatory species also consume rice pollen (Table S3). The following predators are abundant in Chinese rice fields, are exposed to plant‐produced Cry protein, and are available and amenable for testing under controlled laboratory conditions: *Cyrtorhinus lividipennis* (Hemiptera: Miridae), *Chrysoperla nipponensis* (Neuroptera: Chrysopidae), *Propylea japonica* (Coleoptera: Coccinellidae), *Paederus fuscipes* (Coleoptera: Staphylinidae), and the two spiders *Pirata subpiraticus* (Araneae: Lycosidae) and *Ummeliata insecticeps* (Araneae: Linyphiidae) (Table 1). These thus represent a suitable set of test species for initial, early‐tier risk assessment studies.

**Table 1:** Predatory arthropod species recommended as surrogate test species to support the environmental risk assessment of insecticidal GM rice

Species	Order: Family	Food and feeding mode	Studies demonstrating testability of the species
*Cyrtorhinus lividipennis*	Hemiptera: Miridae	Larvae and adults are predators on arthropod herbivores (mainly planthoppers); adults also suck sap of plant leaf or stem when prey is lacking; plant‐dwelling	Han *et al*. (2014)
*Chrysoperla nipponensis* (syn.: *C. sinica*)	Neuroptera: Chrysopidae	Larvae feed on arthropods (mainly aphids) (piercing‐sucking). Adults feed on pollen and nectar. Both stages are plant‐dwelling	Li *et al*. (2014a,b)
*Propylea japonica*	Coleoptera: Coccinellidae	Both larvae and adults feed on arthropods (mainly aphids/planthoppers), and consume pollen during plant anthesis; plant‐dwelling	Zhang *et al*. (2014); Li *et al*. (2015)
*Paederus fuscipes*	Coleoptera: Staphylinidae	Larvae and adults feed on arthropods; soil‐ and plant‐dwelling	Cheng *et al*. (2014)
*Pirata subpiraticus*	Araneae: Lycosidae	Larvae and adults feed on arthropods; soil‐dwelling	Chen *et al*. (2009)
*Ummeliata insecticeps*	Aranea: Linyphiidae	Larvae and adults feed on arthropods; web building	Tian *et al*. (2010)

## Materials and methods

### Identification of the aboveground arthropods in rice

*Bt* rice could be planted in the rice‐growing regions of Central and Southern China. To identify the taxonomic groups and species of aboveground arthropods in those regions, literature searches were conducted. The Web‐of‐Science Core Collection (Thomson Reuters, New York, NY) and the China National Knowledge Infrastructure (CNKI) (Tsinghua Tongfang Knowledge Network Technology Co., Ltd., Beijing, China) were used to cover both international and Chinese peer‐reviewed literature. The following broad search terms were used for retrieving the references from both sources: *rice* and (*arthropod* or *invertebrate* or *predator* or *parasitoid* or *insect*). The collected references were manually screened, and references that fulfilled one of the following criteria were excluded from further analyses: (i) the reference did not contain original data; (ii) the data presented were not from a field survey in rice; (iii) the data were collected in regions other than Central and Southern China (area shaded in red in Figure 1); (iv) the reference contained no data at the species level, or (v) the reference was published in a noncore Chinese academic journal of uncertain quality (high potential for erroneous species identification). The remaining references were used to compile the list of rice arthropods. The total number of records of each species was used as a rough indicator for its abundance in rice fields (Meissle *et al*., 2012; Romeis *et al*., 2014). For each arthropod species, the scientific name and the taxonomic classification (family, order) were confirmed using the Catalogue of Life (http://www.catalogueoflife.org/). For species not included in the Catalogue of Life, databases including the Global Biodiversity Information facility (http://www.gbif.org/), Insektoid.info (http://insektoid.info), or other specialised sources were consulted (Table S1). Species belonging to two functional groups, for example, Syrphidae with predatory larvae and pollinating adults, were assigned to the function rated as most important (e.g., Syrphidae were considered predators).

### Establishment of the arthropod food web

NTAs may be exposed to the plant‐produced insecticidal proteins through various routes, but mainly via consumption of GE plant tissues or by predation or parasitism of herbivores of the GE crop (Raybould *et al*., 2007; Romeis *et al*., 2009). To illustrate the routes by which natural enemies may be exposed to insecticidal proteins in GE rice fields in China, a simplified arthropod food web was constructed. This food web included the most abundant species (species for which ≥10 records were available; Table S1) based on reported trophic links between herbivores and natural enemies from the retrieved literature (Figure 2; Table S2).

### Exposure of rice arthropods to Cry2A produced by *Bt* rice

#### Experimental Bt rice field

In 2011 and 2012, a *Bt* rice line (T2A‐1) expressing a modified *cry2A* gene under the control of the maize ubiquitin promoter (Chen *et al*., 2005) and the corresponding nontransformed near‐isoline Minghui 63 (hereafter referred to as ‘control rice’) were grown in an experimental field in a suburb of Xiaogan City (29.25°N, 108.21°E) in Hubei Province, China (Figure 1). This *Bt* rice line was selected for the field experiment as it contains high concentrations of Cry protein when compared to other *Bt* rice lines (Liu *et al*., 2016). The field was divided into eight plots, and each plot was approximately 109 m^2^. Plots were separated by a nonplanted buffer of 1 m, and the whole field was surrounded by a 1‐m buffer of conventional rice. Both *Bt* rice and control rice were planted in four plots arranged in a randomised block design. The rice seeds were sown in a seeding bed on 25 May 2011 and on 17 May 2012, and the seedlings were transplanted to the experimental plots at the four‐leaf stage (23 June 2011 and 17 June 2012). The plots were cultivated according to the common local agricultural practices, but no insecticide was applied.

#### Collection of arthropods

For determination of the Cry2A concentrations in rice arthropods, the most frequent aboveground arthropod species were collected in the four *Bt* and four control rice plots during (August) and after (September) anthesis in 2011 and before (June), during (August) and after (September) anthesis in 2012. Arthropods were collected by suction sampling, by hand (visual collection), or by using a beating sheet. Plant tissue was also collected on each sampling date; leaf tissue and pollen were collected in 2011, and leaf tissue, pollen and rice stems were collected in 2012. The collected arthropods and plant tissues were immediately placed in a portable refrigerator and transported to the laboratory. After taxonomic identification, individuals (1 to more than 100 depending on size and availability) of the different arthropod species collected on the same date in the same plot were pooled as one replicate and placed in a 5‐mL centrifuge tube. All samples were stored at −80°C before ELISA measurements.

#### ELISA analyses

The concentration of Cry2A was quantified by double‐antibody sandwich enzyme‐linked immunosorbent assays (DAS‐ELISA) using QuantiPlate Kits from EnviroLogix (Portland, ME). Arthropods collected in the field were washed with deionised water to minimise contamination by debris and pollen. All samples of arthropods, rice leaf tissue, stem tissue and pollen were lyophilised before being weighed on an electronic balance (CPA224S, Sartorius, Göttingen, Germany; accuracy = 0.1 ± 0.1 mg). Phosphate‐buffered saline with Tween (PBST) at a ratio of 1:30 to 1:100 mg DW/mL buffer was added to the samples. If sample DW was <3 mg, 300 μL buffer was used (Meissle and Romeis, 2009). For maceration, two 3‐mm tungsten carbide balls were added to each sample, and the samples were shaken for 3 min at 30 Hz in a MM400 mixer mill (Retsch, Haan, Germany) fitted with 24‐tube adapters for microreaction tubes. After centrifugation at 15 800 ***g***, the supernatants were diluted with PBST according to the expected Cry2A concentration. Antibody‐coated plates were loaded with enzyme conjugate and Cry2A standards provided with the kit, negative controls (buffer only) and the diluted sample extracts. After the plates were incubated for 1 h under ambient conditions, they were washed four times with PBST, and the provided substrate solution was added. After 30 min of incubation at ambient temperature, 100 μL of stop solution (1.0 N hydrochloric acid) was added per well. After 15 min of incubation, the optical density (OD) was measured at a light wavelength of 450 nm with a microplate spectrophotometer (PowerWave XS2; BioTek, Winooski, VT, USA). Cry2A concentrations (μg/g DW) were calculated using regression analysis. For the clear separation of positive readings from controls, the LOD of the test was determined based on the standard deviation of the OD values of buffer‐only controls multiplied by three (Meissle and Romeis, 2009). Subsequently, the detection limit of each sample was calculated from the dilution, sample weight and amount of added buffer.

Because arthropod and plant tissue samples from the control rice treatment in the field did not show OD values systematically different from those of buffer‐only controls, no cross‐reaction of arthropod proteins with ELISA was apparent.

## Conflict of interests

The authors declare no conflict of interests.

## Supporting information


**Table S1** Arthropod species recorded in the rice‐growing regions of Central and Southern China.

**Table S2** Trophic interactions between arthropods on rice in Central and Southern China.

**Table S3** Cry2A concentrations in arthropods and plant tissues collected in experimental *Bacillus thuringiensis* (Bt) rice plots in Xiaogan (Hubei Province, China) during and after anthesis in 2011 and before, during and after anthesis in 2012.
